# A multi-site study examining the tobacco withdrawal trajectory in people with tobacco and cannabis co-use

**DOI:** 10.1016/j.drugalcdep.2025.112778

**Published:** 2025-07-03

**Authors:** Rachel A. Rabin, Caryn Lerman, Robert Schnoll, Rachel F. Tyndale, Tony P. George

**Affiliations:** a Department of Psychiatry, McGill University, Montreal, Quebec and Douglas Mental Health University Institute, Montreal, Quebec, Canada; b Department of Psychiatry and Norris Cancer Center, University of Southern California, Los Angeles, CA, USA; c Department of Psychiatry and Abramson Cancer Center, University of Pennsylvania, Philadelphia, PA, USA; d Campbell Family Mental Health Research Institute, Centre for Addiction and Mental Health, University of Toronto, Toronto, ON, Canada; e Department of Pharmacology and Toxicology, University of Toronto, Toronto, ON, Canada; f Department of Psychiatry, University of Toronto, Toronto, ON, Canada; g Addictions Division and Institute for Mental Health Policy and Research, Centre for Addiction and Mental Health, Toronto, ON, Canada

**Keywords:** Tobacco, Nicotine, Cigarettes, Co-use, Cannabis, Tobacco withdrawal, Abstinence

## Abstract

**Background:**

Approximately 30 % of people who use tobacco also use cannabis, and rates of co-use are rising. Relative to people who use tobacco alone (TO), individuals who co-use tobacco and cannabis (TC) experience greater difficulty with tobacco cessation, yet mechanisms underlying this phenomenon remain unexplored. Leveraging data from a multi-site, double-blind clinical trial for tobacco cessation, we compared the trajectory of tobacco withdrawal, a strong predictor of relapse, between TC and TO during 11-weeks of tobacco treatment.

**Methods:**

People seeking treatment for tobacco were randomized to one of three arms (placebo, nicotine patch or varenicline) and followed for 11-weeks. Participants were parsed according to their cannabis use status determined by a cannabis-positive urine toxicology at screen (N = 1246). We selected participants with end-of-treatment biochemically verified 7-day point prevalence tobacco abstinence (N = 330; TC, n = 55 and TO, n = 275) and examined group differences in tobacco withdrawal severity using the Minnesota Nicotine Withdrawal Scale (MNWS) at baseline, week 1, 4, 8, and week 11 (end-of-treatment).

**Results:**

Controlling for age, treatment arm, and site, we found a significant interaction (group × time) effect for withdrawal severity (p < 0.01). Bonferroni-corrected post-hoc comparisons revealed that relative to TO, TC had elevated withdrawal scores at week 1 (TC, M=9.3 ± 5.5; TO, M=7.1 ± 5.6; p < 0.01); no other timepoints showed between-group differences.

**Conclusions:**

People who co-use experience greater tobacco withdrawal severity one-week post abstinence compared to people who only use tobacco. Personalized interventions that target immediate tobacco withdrawal and/or cannabis use may help improve tobacco cessation rates for people who co-use both substances.

## 1. Introduction

Tobacco and cannabis are among the most widely used substances globally, and co-use of these substances is increasing by ~2 % per year, a trend that has been apparent over the past two decades (Goodwin et al., 2018; Rubenstein et al., 2024; Weinberger et al., 2022b). Higher rates of cannabis use are consistently observed in those who smoke tobacco cigarettes compared to people who do not smoke cigarettes (Weinberger et al., 2022a) with estimates indicating that up to 30 % of those using tobacco are co-using cannabis (Gravely et al., 2022, 2020).

Tobacco and cannabis co-use poses substantial health challenges, given their combined adverse health effects and treatment consequences. Research indicates that co-use is associated with additive exposure to toxicants and an increased risk of developing mental health disorders (Do et al., 2024; Meier and Hatsukami, 2016; Rabin et al., 2023). Additionally, co-use is linked to higher tobacco and cannabis consumption as well as increased dependence severity for each substance (Hindocha et al., 2015; Rabin and George, 2015; Weinberger et al., 2018).

Although the evidence is mixed, several studies suggest that cannabis co-use is associated with poorer tobacco cessation outcomes, which is highly concerning given that cigarette smoking remains the leading cause of preventable disease and premature death in the United States (Creamer et al., 2019). Notably, several epidemiological studies have demonstrated that cannabis co-use is associated with a negative impact on tobacco cessation outcomes (Driezen et al., 2022; El-Khoury Lesueur et al., 2018; Voci et al., 2020; Weinberger et al., 2020). Similarly, data from individuals seeking treatment for tobacco via quit lines showed that people who co-used cannabis were less likely to successfully quit tobacco compared to people without cannabis co-use (Zhu et al., 2024) and compared to people with non-daily cannabis use (Goodwin et al., 2022). Lastly, several secondary analyses of randomized clinical trials (RCT) evaluating pharmacotherapies (e.g., varenicline, nicotine patch) found that people with cannabis co-use had reduced odds of tobacco abstinence compared to people without cannabis co-use (McClure et al., 2020a; Ozga et al., 2023; Rogers et al., 2020). Although not all RCT report this association (McClure et al., 2020b; Metrik et al., 2011; Rabin et al., 2016; Walsh et al., 2020). Importantly, mechanisms underlying poorer treatment outcomes among people who co-use, relative to tobacco-only use, have not yet been identified.

Notably, people who use cannabis and co-use tobacco have lower rates of cannabis abstinence compared to people with cannabis-only use (Gray et al., 2017; Haney et al., 2013). Further, there is burgeoning evidence that people who co-use are more likely to experience cannabis withdrawal symptoms, experience them at greater severity and for a longer duration compared to people with cannabis-only use (Bahji et al., 2020; Preuss et al., 2010; Yeap et al., 2024, 2023). Notably, prospective studies demonstrate that cannabis withdrawal severity increases during periods of cannabis abstinence, even when tobacco use remains stable, indicating that these effects are specifically attributable to cannabis cessation rather than the cessation of both substances (Preuss et al., 2010; Yeap et al., 2024, 2023). Thus, worse treatment outcomes associated with co-use may reflect elevated and prolonged cannabis withdrawal (Allsop et al., 2012; Chung et al., 2008; Greene and Kelly, 2014; Haney, 2005).

Given that tobacco withdrawal predicts relapse (Robinson et al., 2019), we tested for the first time whether following tobacco cessation, tobacco withdrawal symptoms parallel the trajectory observed with cannabis withdrawal symptoms following cannabis cessation. We also assessed affective-motivational processes, including craving and positive and negative affect, to determine whether co-use effects were specific to tobacco withdrawal or reflected broader emotional and motivational dysregulation.

Leveraging data from a multi-site, double-blind clinical trial for tobacco cessation (Lerman et al., 2015), we compared the trajectory of tobacco withdrawal between people with tobacco and cannabis co-use and people with tobacco-only use during 11-weeks of tobacco treatment. Results may inform our understanding of how tobacco withdrawal functions as a key mechanism through which co-use of cannabis adversely diminishes the effectiveness of tobacco cessation outcomes.

## 2. Material and methods

### 2.1. Participants

We used data from a completed multi-site tobacco treatment trial (NCT01314001) (Lerman et al., 2015). The institutional review boards at all four sites (University of Pennsylvania, Centre for Addiction and Mental Health, SUNY Buffalo, MD Anderson Cancer Center) approved the protocol. All participants provided written, informed consent.

Participants were between 18 and 65 years old, were treatment-seeking for tobacco, and smoked ≥ 10 cigarettes per day for 6 months or longer (verified by carbon monoxide concentrations >10 ppm).

Trial exclusion criteria included use of non-cigarette tobacco products, electronic cigarettes, or current tobacco treatment; history of substance misuse treatment, current use of cocaine or methamphetamine, or > 25 alcoholic drinks per week; medical contraindications (pregnancy, history of cancer, kidney or liver disease, or transplant, clinically significant cardiac dysrhythmias, stroke, angina, heart attack, or uncontrolled hypertension); history of an Axis 1 psychiatric disorder or suicide risk score on the Mini International Neuropsychiatric Interview of > 1; current use of antipsychotics, stimulants, opiate medications, anticoagulants, rescue inhalers, antiarrhythmics, or medications altering CYP2A6 activity or any condition that could compromise safety. Additionally, we excluded participants who had missing cannabis toxicology data and those that had missing data for all 5 MNWS timepoints (baseline, week 1, week 4, week 8, and week 11).

A total of 1246 participants completed the trial. For our secondary analysis, we selected participants with end of treatment (week 11) biochemically verified 7-day point prevalence tobacco abstinence to control for the effects of smoking on self-reported withdrawal. Abstinence was defined as no self-reported smoking (not even a puff) for at least 7 days before the week 11 telephone assessment, with in-person verification for those self-reporting abstinent (carbon monoxide ≤8 ppm). For robustness, we re-ran analyses that included participants that did not meet this criterion (i.e., non-abstinent participants).

Participants with positive urine assays for 11-nor-9-carboxy-delta-9-tetrahydrocannabinol (THC-COOH >50 ng/mL) at the screening visit were categorized as individuals with tobacco-cannabis use (TC), and those with negative results (THC-COOH <50 ng/mL) were categorized as individuals with tobacco-only use (TO).

### 2.2. Study procedures

After eligibility was confirmed, individuals were randomly assigned by baseline 3ʹ-hydroxycotinine:cotinine (NMR) status and study site in blocks of 12 patients (1:1:1 ratio), to 11 weeks of placebo (placebo pill plus placebo patch), nicotine patch (active patch plus placebo pill), or varenicline (active pill plus placebo patch). All participants received behavioral counseling which focused on skills to quit and avoid relapse, instructions on the use of medication, and medication compliance. Participants and investigators were blind to group allocation and NMR status. For a full description of procedures please see Lerman et al. (2015).

### 2.3. Measures

At screening, participants completed demographic assessments, clinical interviews (e.g., Mini International Neuropsychiatric Interview) and smoking-related questionnaires (e.g., timeline follow-back, Fagerstrom Test for Nicotine Dependence). At baseline, participants provided plasma samples to estimate the rate of nicotine clearance using the nicotine metabolite ratio (NMR, 3-hydroxycotinine/cotinine) and were classified as normal or slow nicotine metabolizers using cut-points established (see Lerman et al., 2015).

Nicotine withdrawal symptom severity was measured using the Minnesota Nicotine Withdrawal Scale (MNWS) (Hughes et al., 1984). Each of the 15 items (e.g., anxiety, irritability, craving, nausea) was rated on a scale of 0–4 with 0 =none, 1 = Slight, 2 = Mild, 3 = Moderate, and 4 =Severe. A total score was calculated by summing all items.

As secondary analyses, we sought to determine whether craving and affect followed similar trajectories as withdrawal. Therefore, we included assessments of craving using the Brief Questionnaire of Smoking Urges (QSU-B) (Cox et al., 2001), and positive and negative affect using the Positive and Negative Affect Schedule (PANAS) (Watson et al., 1988). The QSU-B is a 10-item questionnaire that employs a 7-point Likert scale ranging from “Strongly Disagree” to “Strongly Agree.” The PANAS is a 20-item self-report questionnaire that measures positive and negative affect using a 5-point Likert scale ranging from “Very slightly or not at all” to “Extremely.” All three assessments were completed at the target quit date (week 0), and at the end of weeks 1, 4, 8, and 11. The PANAS and MNWS assessed symptoms “during the last week,” and the QSU-B assessed craving “right now.”

### 2.4. Statistical analysis

Statistical analyses were performed using SPSS Statistics 29.0. Group differences in demographic, substance use, and clinical variables were analyzed using chi square tests for categorical variables and independent t-tests for continuous variables.

To determine group differences in the severity of tobacco withdrawal symptoms (MNWS score) during treatment (baseline, week1, week 4, week 8, week 11), we conducted a generalized linear mixed model (GLMM) that included the interaction term group × time. The model was adjusted for age, site, and treatment arm, and included participant identification number as a random effect. Significant interaction effects were followed-up with Bonferroni-corrected post-hoc comparison. To test whether group (TC versus TO) predicted smoking abstinence at week 11, a binary logistic regression was conducted controlling for site, age and treatment arm. We also reran the GLMM including non-abstinent participants to test whether the results remained consistent when non-abstinent individuals were included. For our secondary analyses, which explored craving and affect trajectories, we conducted three additional GLMM analyses using the QSU-B, PANAS positive affect score, and PANAS negative affect score as dependent variables. Statistical significance was set at α = .05 for all analyses.

## 3. Results

### 3.1. Participants

A total of 1246 participants were enrolled in the study. Twenty participants had missing cannabis toxicology data, and 33 participants had missing MNWS data at all 5 timepoints; these participants were excluded (N = 1193). Of these, 330 met end of trial tobacco abstinence and were classified as TC (n = 55) or TO (n = 275). Demographic and substance use data for the TC and TO groups are summarized in Table 1. The groups were comparable on sex, race and employment status. Additionally, the TC and TO groups did not differ on NMR status, average daily cigarettes, nicotine dependence severity score, or average alcoholic drinks consumed per week. Lastly, the distribution of participants across treatment arms (placebo, nicotine patch and varenicline) was similar between TC and TO groups. However, age differed between groups with TC [*M*= 41.9 (10.3)] being significantly younger than the TO group [*M*= 47.5 (10.3)]; age was therefore included as a covariate in all analyses.

### 3.2. Tobacco withdrawal severity

#### 3.2.1. Abstinent participants

Across all 5 time-points, there were 24 timepoints that had missing MNWS data: baseline (n = 8); week 1 (n = 3); week 4 (n = 5); week 8 (n = 8) and week 11 (n = 0).

**Fig. 1.**
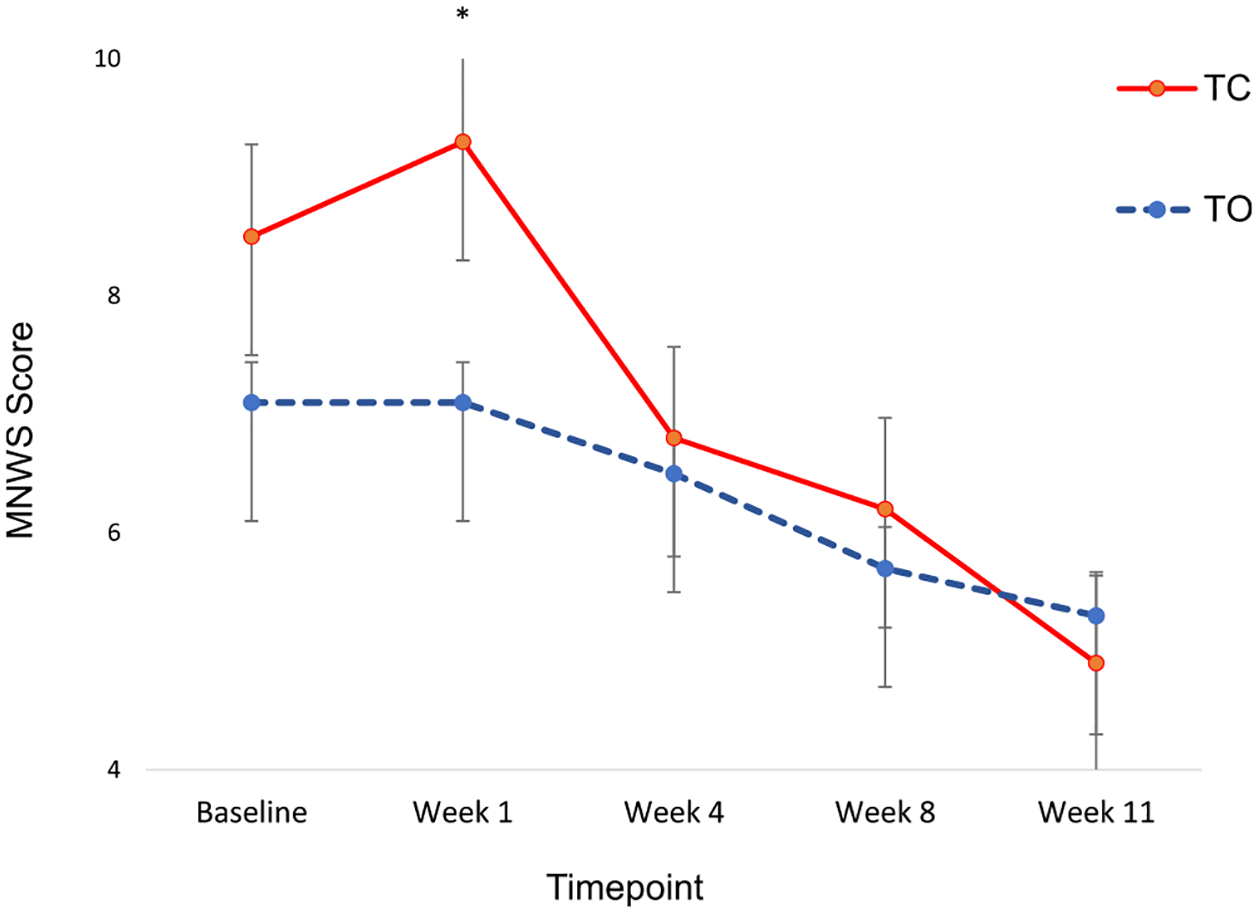
Between Group Differences in Tobacco Withdrawal Severity During Abstinence. There was a significant interaction between group and time on tobacco withdrawal severity. A significant group difference in MNWS score was found at week 1 (p < 0.01), such that TC had greater withdrawal severity than TO. No additional between-group differences were observed at any other timepoint. * p < 0.01. MNWS, Minnesota Nicotine Withdrawal Scale; TC, people with cannabis-tobacco co-use; TO, people with tobacco-only use.

A GLMM revealed a significant main effect of time [*F*(4, 1610) = 21.52, *p* < 0.01], indicating that withdrawal severity varied across the abstinence period, with the highest scores observed at early time points. While the main effect of group was not significant [*F*(1, 1610) = 1.27, *p* = 0.26], a significant interaction between time and group emerged [*F*(4, 1610) = 3.63, *p* < 0.01)]. Post-hoc pairwise comparisons revealed that at week 1, TC reported significantly greater withdrawal severity compared to TO [*Mean difference* = 2.28, *SE* = 0.82, *t*(1610) = 2.79, *p* = 0.005, d= 0.41)]. Group differences were not observed at any other timepoint. See Fig. 1. Means and standard deviations of the MNWS scores at each timepoint are provided in Table 2. As a sensitivity analysis, the models were rerun with NMR as a covariate, and the results remained unchanged.

Lastly, a binary logistic regression was conducted to examine whether group (TC versus TO) predicted smoking abstinence at end of treatment. The model was not statistically significant, χ^2^(1) = 0.41, p = 0.89.

#### 3.2.2. Abstinent + non-abstinent participant*s*

Of the 1193 in the final sample, n = 215 were classified as TC, and n = 978 as TO. Given that age, sex, and alcoholic drinks per week differed between groups (See Table S1), we included these variables as fixed effects in the model. Notably, when non-abstinent participants were included in the model, the significance of the group × time effect reduced to a trend [F (4,5019) = 2.01, p = 0.080]. However, the overall pattern of results remained the same, see Figure S1.

### 3.3. Craving trajectories

**Fig. 2.**
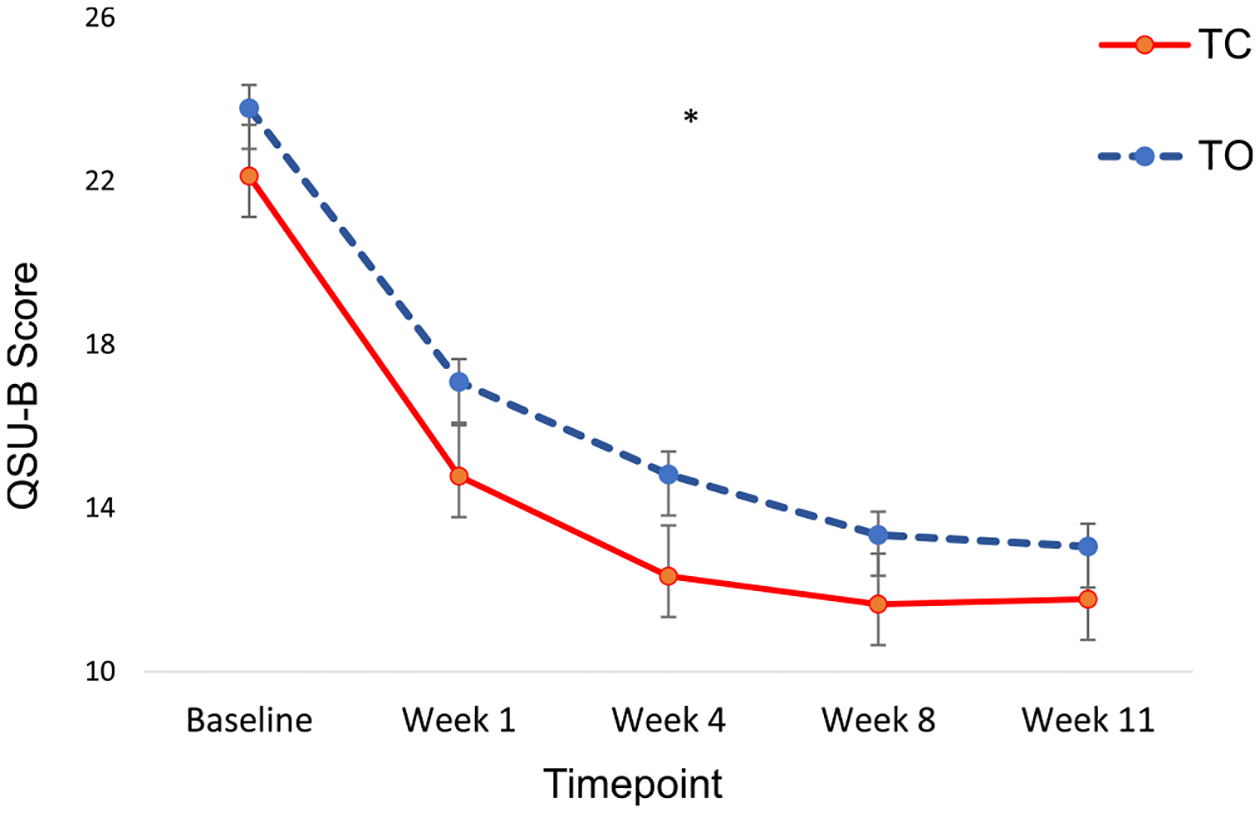
Between Group Differences in Tobacco Craving Severity During Abstinence. Both the TC and TO groups decreased linearly during abstinence. The interaction between time × group was not significant. * p < 0.05 main effect of time. QSU-B, Brief Questionnaire of Smoking Urges; TC, people with cannabis-tobacco co-use; TO, people with tobacco-only use.

For the QSU-B, results revealed a significant main effect of time F (4,1610) = 70.28, p < 0.01] depicting decreasing severity scores over time for both groups. The group effect (p = 0.06) and interaction effect (group × time) were not significant, *p* = 0.92. See Fig. 2.

### 3.4. Positive and negative affect trajectories

Both PANAS positive and negative affect scores remained stable over time (p’s > 0.05), with no significant group or group × time effects (p’s > 0.05).

## 4. Discussion

This is the first study to investigate the effects of cannabis co-use on the tobacco withdrawal trajectory during tobacco treatment. Among individuals with tobacco-only use, tobacco withdrawal severity decreased during 11-weeks of treatment, in line with what has been reported in the literature (Hughes, 2007; McLaughlin et al., 2015). In contrast, among individuals with cannabis co-use, withdrawal severity increased from baseline to week one, where it peaked, followed by a decline over the subsequent 10 weeks of treatment. Notably, tobacco withdrawal severity at baseline was comparable between groups; similarly, there were no group differences following 4, 8, and 11 weeks of abstinence and treatment. This indicates that group differences in withdrawal severity only emerge within the first week of tobacco abstinence, underscoring that cannabis co-use contributes to a more problematic withdrawal trajectory. While the effect size for the group difference in tobacco withdrawal severity at week 1 was modest (d = 0.41), the findings still hold clinical relevance as even small increases in withdrawal severity have been associated with poorer treatment outcomes, distress, and interruption of daily functioning (al’Absi et al., 2004; Conti et al., 2020). Of note, cannabis co-use was not associated with lower abstinence rates at end of treatment, consistent with our prior analysis of the same dataset conducted in an independent subset of participants matched on age and sex (Rabin et al., 2016). Nevertheless, this was surprising given the role withdrawal symptoms play in driving continued substance use, reducing treatment adherence, and increasing relapse risk (Catz et al., 2011; Kenny and Markou, 2001). Lastly, the effects of cannabis co-use were specific to tobacco withdrawal and did not extend to other affective-motivational processes, such as craving, positive affect or negative affect.

Rerunning the analyses to include non-abstinent participants, reduced the significance of the interaction between group and time on withdrawal severity scores to a trend. This may reflect that smoking attenuates withdrawal severity, highlighting that cannabis co-use exacerbates tobacco withdrawal particularly under conditions of abstinence. Therefore, identifying the mechanism facilitating cannabis’ effect on tobacco withdrawal during tobacco abstinence may help guide the development of tailored treatments for this specialized but prevalent population.

First, it is unlikely that the association between cannabis co-use and increased withdrawal is driven by baseline differences in CYP2A6 activity, the major nicotine- and cotinine-metabolizing enzyme (Dempsey et al., 2004) given that NMR status did not differ between groups. This is important to rule out since it has been demonstrated that fast metabolizers experience greater tobacco withdrawal during abstinence relative to slow metabolizers (Liakoni et al., 2019; Rubinstein et al., 2008; Sofuoglu et al., 2012).

However, it is possible that elevated tobacco withdrawal severity associated with cannabis co-use may involve crosstalk between the endogenous nicotinic and cannabinoid systems (Valjent et al., 2002). Notably, tobacco withdrawal has been linked to the upregulation of nicotinic acetylcholine receptors (nAChR) containing the β2 subunit (Jackson et al., 2008; Simmons and Gould, 2014). Moreover, evidence suggests that people with co-use have greater β2 nAChR availability compared to people with tobacco-only use (Brody et al., 2016). Thus, the increased presence of the β2 nAChR subunit may be driving elevated withdrawal severity among people with co-use, although this has not been empirically tested.

Endocannabinoid signaling may also contribute to elevated tobacco withdrawal symptoms. Anandamide and 2-arachidonoylglycerol (2-AG) are two prominent endocannabinoids and the enzymes that degrade them are fatty acid amide hydrolase (FAAH) and monoacylglycerol lipase (MAGL), respectively. Preclinical studies demonstrate associations between low levels of 2-AG and more severe somatic withdrawal symptoms (Muldoon et al., 2015; Saravia et al., 2017). Similarly, inhibiting FAAH, which increases anandamide, prevented the anxiogenic-like responses associated with nicotine abstinence (Cippitelli et al., 2011). Consistent with these findings, studies in humans have reported negative relationships between endocannabinoids and depressive and anxiety symptoms (Gao et al., 2020; Hill et al., 2008; Lazary et al., 2021), which are typical tobacco withdrawal symptoms (Hughes, 2007). Given that both tobacco use and cannabis use are independently associated with lower endocannabinoid levels (Gonzalez et al., 2002; Hirvonen et al., 2018; Morgan et al., 2013), their combined use may have an additive impact on endocannabinoid signaling, potentially intensifying tobacco withdrawal symptoms. Thus, individuals who co-use cannabis and tobacco may have lower endocannabinoid levels compared to those who use tobacco alone.

Our results have important clinical implications for improving tobacco cessation outcomes for people with co-use, a subgroup that is steadily increasing (Rubenstein et al., 2024). Indeed, heightened tobacco withdrawal severity may help explain the elevated relapse rates among people with co-use relative to people with tobacco-only use (Driezen et al., 2022; El-Khoury Lesueur et al., 2018; McLaughlin et al., 2015; Voci et al., 2020; Weinberger et al., 2020). This knowledge can equip treatment providers with awareness of when people with co-use are at high risk for relapse so that effective intervention can be delivered during this sensitive period. Moreover, tailored interventions that target the unique withdrawal challenges of people with co-use may be essential for improving smoking cessation outcomes for this population. Alternatively, interventions that simultaneously target cannabis use may help optimize successful tobacco cessation.

The present study has several limitations. First, cannabis use was indexed via urine toxicology and thus groups were categorized based on a binary THC-COOH cut-off, which provides little information regarding cannabis use patterns. Further, participants meeting criteria for cannabis use disorder were excluded, precluding investigation of people with problematic cannabis use. Given that no data was collected regarding cannabis use at baseline as well as under conditions of abstinence, it is unknown if in this sample tobacco abstinence exerts a compensatory effect on cannabis use. Therefore, it would be important to record cannabis consumption during tobacco abstinence. In fact, other studies report that reductions in cannabis use may accompany tobacco abstinence (Metrik et al., 2011; Nguyen et al., 2022). Thus, dual abstinence from both cannabis and tobacco may have contributed to the observed heightened tobacco withdrawal severity since cannabis withdrawal symptoms overlap with symptoms of tobacco withdrawal (Vandrey et al., 2008). An additional limitation is that tobacco withdrawal assessments were only done at week 1, 4, 8, and 11. Given that severity of tobacco withdrawal typically peaks in the first 24–72 h (Hughes, 2007), assessing group differences before week one would provide valuable insights into the early dynamics of withdrawal severity between people with co-use relative to tobacco-only use. Similarly, it would be important to determine, among people with co-use, when during the first and fourth week of abstinence withdrawal severity begins to decline. Participants in this study were randomized to different pharmacotherapies (nicotine patch, varenicline) or assigned to the placebo arm. While we controlled for treatment arm, variations in treatment response may have influenced withdrawal severity. Lastly, data for this study were collected between 2010 and 2013. Since then, laws surrounding cannabis use have relaxed, cannabis product types and modes of consumption have diversified, and the potency of tetrahydrocannabinol, the main psychoactive ingredient in cannabis, has increased substantially (ElSohly et al., 2021; Hammond et al., 2022, 2020). As a result, study findings may not fully generalize to the current cannabis landscape.

In summary, our findings suggest that cannabis co-use significantly worsens the severity of tobacco withdrawal symptoms following tobacco cessation at one-week post-abstinence. Given that withdrawal is a major contributor of relapse, findings from this study might help inform the development of targeted treatments for the growing population of people with co-use. Personalized therapies that target both tobacco withdrawal and cannabis use may be the most effective approach for optimizing successful cessation in individuals with co-use (Rabin, 2020). Future studies should include participants with heavier cannabis use (e. g. cannabis use disorder), record patterns of cannabis use during tobacco abstinence, compare endocannabinoid levels between people with co-use and tobacco-only use, and assess withdrawal more frequently within the first month of tobacco abstinence. Such methodological advancements can further guide interventions to support sustained tobacco abstinence among individuals who co-use both tobacco and cannabis.

## Supplementary Material

1

## Figures and Tables

**Table 1:** Demographic and substance use characteristics in abstinent participants.

	TC (n = 55)	TO (n = 275)	*p*-value
**Age**	**41.9 (10.8)**	**47.5 (10.3)**	**< 0.01**
Sex (M/F)^n^	34/21	155/120	0.46
Race: White^n^ (%)	32 (58 %)	166 (60 %)	0.76
Race: Black^n^ (%)	23 (42 %)	97 (35 %)	0.36
Employment Status (FT/PT/not working)^n^	32/5/18	146/38/91	0.60
NMR status (Normal/Slow)	26/29	139/136	0.66
Treatment Arm (PL/NP/Var)^n^	13/16/26	62/93/120	0.79
Average cigarettes per day	18.9 (9.2)	17.4 (7.4)	0.21
FTND	4.9 (2.3)	4.7 (2.1)	0.38
Average alcoholic drinks per week	3.8 (6.0)	3.3 (5.6)	0.59
Baseline QSU-B	21.8 (11.1)	23.4 (13.5)	0.41
Baseline PANAS-P	31.8 (9.9)	34.4 (8.9)	0.07
Baseline PANAS-N	14.0 (5.7)	13.2 (4.2)	0.20

Values given in mean (standard deviation), except for variables denoted with “n” sex, where number of participants are provided.

F, female; FTND, Fagerstrom Test for Nicotine Dependence; FT, Full-time work; M, Male; MNWS, Minnesota Nicotine Withdrawal Scale; NMR, 3ʹ-hydroxycotinine: cotinine; PANAS-N, Positive and Negative Affect Schedule Negative Subscale; PANAS-P, Positive and Negative Affect Schedule Positive subscale; PL, placebo; PT, Part-time work; NP, nicotine patch; QSU-B, Questionnaire of Smoking Urges Brief; TC, individuals with tobacco-cannabis co-use; TO, individuals with tobacco-only use; Var, varenicline

**Table 2:** Means and standard deviations of MNWS scores in abstinent participants.

	TC (n = 55)	TO (n = 275)
Baseline	8.53 (5.6)	7.1 (5.6)
**Week 1** *	**9.3 (5.5)**	**7.1 (5.6)**
Week 4	6.8 (5.5)	6.5 (5.6)
Week 8	6.2 (5.5)	5.7 (5.7)
Week 11	4.9 (5.5)	5.3 (5.6)

* Bonferroni-adjusted p-value < 0.05

## Appendix A. Supporting information

Supplementary data associated with this article can be found in the online version at doi:10.1016/j.drugalcdep.2025.112778.
