# Supplementary material for: A multi-site study examining the tobacco withdrawal trajectory in people with tobacco and cannabis co-use
Source: Drug Alcohol Depend. Author manuscript; Available in PMC 2026 Jul 1. (PMC13322613; doi:10.1016/j.drugalcdep.2025.112778)
Supplement: 1 [file NIHMS2169367-supplement-1.docx]

**Table S1: Demographic and Substance Use Characteristics in the Full Sample**

|  | **TC (n=215)** | **TO (n=978)** | ***p*-value** |
| --- | --- | --- | --- |
| **Age** | **39.88 (11.7)** | **46.7 (10.8)** | **<0.01** |
| **Sex (M/F) ^n^** | **76/139** | **448/530** | **<0.01** |
| Race: White^n^ (%) | 119 (55%) | 558 (57%) | 0.65 |
| Race: Black^n^ (%) | 86 (40%) | 376 (40%) | 0.67 |
| Employment Status (FT/PT/not working)^n^ | 97/36/82 | 447/163/368 | 0.99 |
| NMR status (Normal/Slow) | 100/115 | 494/484 | 0.29 |
| Treatment Arm (PL/NP/Var) ^n^ | 69/61/85 | 319/341/318 | 0.09 |
| Average cigarettes per day | 17.6 (7.1) | 18.4 (7.3) | 0.14 |
| FTND | 5.1 (2.0) | 5.2 (2.0) | 0.31 |
| **Average alcoholic drinks per week** | **4.0 (5.6)** | **3.0 (4.9)** | **<0.01** |
| Baseline MNWS | 8.30 (5.4) | 8.10 (5.7) | 0.59 |
| Baseline QSU-B | 29.0 (13.9) | 29.8 (14.8) | 0.47 |
| **Baseline PANAS-P** | **32.6 (9.3)** | **34.1 (9.1)** | **0.04** |
| Baseline PANAS-N | 13.6 (4.4) | 13.6 (4.9) | 0.94 |

Values given in mean (standard deviation), except for variables denoted with “n” sex, where number of participants are provided.

F, female; FTND, Fagerstrom Test for Nicotine Dependence; FT, Full-time work; M, Male; MNWS, Minnesota Nicotine Withdrawal Scale; NMR, 3ʹ-hydroxycotinine:cotinine; PL, placebo; PT, Part-time work; NP, nicotine patch; TC, individuals with tobacco-cannabis co-use; TO, individuals with tobacco-only use; Var, varenicline.

**Figure S1 Between Group Differences in Tobacco Withdrawal Severity During Abstinence in the Full Sample**

MNWS, Minnesota Nicotine Withdrawal Scale; TC, people with cannabis-tobacco co-use; TO, people with tobacco-only use.

**Figure S1. Between Group Differences in Tobacco Withdrawal Severity During Abstinence in the Full Sample**

Notably, when non-abstinent participants were included in the model, the significance of the group x time effect reduced to a trend [F (4,5019) = 2.01, p=0.080], however, the overall trajectory remained consistent with the findings observed when only abstinent participants were included.
